# Galectin‐3 and suppression of tumorigenicity 2: Two emerging cardiac biomarkers that may be predictors of cardiac fibrosis development in sport

**DOI:** 10.1113/EP091428

**Published:** 2023-08-28

**Authors:** Caroline Le Goff

**Affiliations:** ^1^ Clinical Chemistry Department University Hospital of Liege Liege Belgium

**Keywords:** cardiac fibrosis, endurance, Galectin‐3, sport, suppression of tumorigenicity 2

1

In an article in this issue of Experimental Physiology, Kröpfl et al. (2023) has  shown that Galectin‐3 (Gal‐3) and Suppression of Tumorigenicity‐2 (ST2) levels increase after acute exercise of different types (cycling and running) (Kröpfl et al., 2023). In this viewpoint, I will share our experience about those biomarkers in sport. In recent years, endurance and ultra‐endurance events have grown in popularity. The human body's capacity to adapt to extremely strenuous physical activity is tested during these events. Intensive exercise causes multi‐organ stress that is underlined by a significant rise in cardiac and inflammatory biomarkers as well as muscular cytolysis (Le Goff et al., 2020). Unfortunately, a variety of earlier investigations have shown death related to cardiac events during or after exercise (Le Goff et al., 2012). A recent systematic study also shows that endurance athletes have a higher prevalence of cardiac fibrosis (Van de Schoor et al., 2015). Intense exercise‐induced cardiac fibroid deposits may contribute to cardiac arrhythmias. Gal‐3 and ST2 are thought to be indicators of fibrosis and cardiac remodelling processes. These proteines are involved in the pathogenesis of heart failure, and people with cardiac fibrosis and remodelling have higher quantities of these two proteins, which may be helpful in tracking the course of the disease. Cardiovascular macrophages that have been stimulated by an inflammatory process release Gal‐3. It is implicated in the recruitment and proliferation of inflammatory cells as well as the activation of cardiac fibroblasts in the case of heart failure, where inflammation appears to play a significant role. Its paracrine and endocrine effects cause pro‐collagen to be secreted, which in turn causes cardiac fibrosis and the subsequent remodelling of the myocardium (Bayes‐Genis et al., 2013; Le Goff et al. 2020). The American College of Cardiology (ACC) and the American Heart Association (AHA) approved the use of Gal‐3 levels as a measure of cardiac prognosis in the early months of 2013. As a measure of inflammation, tissue fibrosis, matrix remodelling and myocyte strain, ST2, often referred to as soluble interleukin‐1 receptor‐like, plays a significant role in cardiovascular disease (Le Goff et al., 2020). According to recent research, ST2 is a new biomarker for predicting heart failure. The American Association of Clinical Chemistry (AACC) and AHA guidelines from 2013 propose measuring ST2 for additive risk stratification in patients with acute or chronic ambulatory heart failure. Heart disease and inflammatory disorders both have higher blood concentrations of ST2, which is regarded as a useful prognostic marker for both conditions. However, at present, according to some studies, ST2 could lack disease specificity and so, is not widely used in routine for the diagnosis of heart failure (Dudek et al., 2020). It is still unknown what the main source of circulating ST2 is in healthy people and patients with various disorders (Le Goff et al., 2020). Gal‐3 and ST2 may therefore be useful for researching cardiac fibrosis and remodelling in athletes in this setting. Studies have shown a substantial increase in the biomarkers for necrosis, strain and inflammation. The possibility of the release of cardiac biomarkers being momentary and so reversible has already been established (Salvagno et al., 2014). It is unknown if sustained, frequent, intense exercise can cause fibrosis and heart failure over the long run. Considering that hormonal imbalance is known to occur in heart failure, ST2 and Gal‐3, which are involved in this process, may offer useful insight as they also increase in these circumstances as demonstrated by Kropfl et al. (2023). We expected low values of these biomarkers in athletes since high plasma levels of Gal‐3 and ST2 are thought to be risk predictors in the general population and for patients with heart failure (Bayes‐Genis et al., 2013). However, recent studies have highlighted that repeated bouts of acute, high‐intensity exercise lead to deleterious changes in heart tissue that may manifest as an abrupt change in cardiac biomarkers, and ST2 and Gal‐3 may thus provide significant information (Le Goff et al., 2020). The emergence of novel cardiac biomarkers Gal‐3 and ST2 could now provide the opportunity to explore which athletes may be most at risk of experiencing sudden cardiac death (SCD), since cardiac fibrosis could contributed to the development of SCD and Gal‐3 and ST2 could give additional information compared with other well‐established biomarkers.

## AUTHOR CONTRIBUTIONS

Sole author.

## CONFLICT OF INTEREST

None declared.

### FUNDING INFORMATION

No funding was received for this work.
